# Combining imaging mass spectrometry and immunohistochemistry to analyse the lipidome of spinal cord inflammation

**DOI:** 10.1007/s00216-024-05190-3

**Published:** 2024-02-07

**Authors:** Ibai Calvo, Alejandro Montilla, Cristina Huergo, Lucía Martín-Saiz, Javier Martín-Allende, Vanja Tepavcevic, María Domercq, José A. Fernández

**Affiliations:** 1 Department of Physical Chemistry, Faculty of Science and Technology, University of the Basque Country (UPV/EHU), Bº Sarriena s/n, 48940 Leioa, Spain; 2 Achucarro Basque Center for Neurosciencie, Bº Sarriena s/n, 48940 Leioa, Spain; 3 Department Neuroscience, Faculty of Medicine, University of the Basque Country (UPV/EHU), Bº Sarriena s/n, 48940 Leioa, Spain; 4 Department of Languages and Computer Systems, School of Engineering, University of the Basque Country (UPV/EHU), Paseo Rafael Moreno “Pitxitxi”, n. 2/3, 48013 Bilbao, Spain

**Keywords:** Lipidomics, Imaging mass spectrometry, Inflammation, Immunohistochemistry

## Abstract

Inflammation is a complex process that accompanies many pathologies. Actually, dysregulation of the inflammatory process is behind many autoimmune diseases. Thus, treatment of such pathologies may benefit from in-depth knowledge of the metabolic changes associated with inflammation. Here, we developed a strategy to characterize the lipid fingerprint of inflammation in a mouse model of spinal cord injury. Using lipid imaging mass spectrometry (LIMS), we scanned spinal cord sections from nine animals injected with lysophosphatidylcholine, a chemical model of demyelination. The lesions were demonstrated to be highly heterogeneous, and therefore, comparison with immunofluorescence experiments carried out in the same section scanned by LIMS was required to accurately identify the morphology of the lesion. Following this protocol, three main areas were defined: the lesion core, the peri-lesion, which is the front of the lesion and is rich in infiltrating cells, and the uninvolved tissue. Segmentation of the LIMS experiments allowed us to isolate the lipid fingerprint of each area in a precise way, as demonstrated by the analysis using classification models. A clear difference in lipid signature was observed between the lesion front and the epicentre, where the damage was maximized. This study is a first step to unravel the changes in the lipidome associated with inflammation in the context of diverse pathologies, such as multiple sclerosis.

**Graphical abstract:**

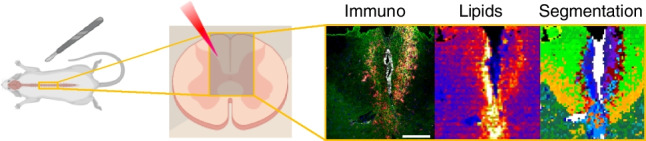

**Supplementary Information:**

The online version contains supplementary material available at 10.1007/s00216-024-05190-3.

## Introduction

Inflammation is a complex process, usually triggered by tissue injury [1]. Despite it is intended to be a protective mechanism, under some circumstances, it can have deleterious effects, giving rise to different pathologies. Among the diseases associated to an incorrect function of the inflammation mechanism, multiple sclerosis has a high prevalence. Multiple sclerosis is an immune-mediated disease in which misdirected T cells, B cells and other inflammatory drivers infiltrate the brain and destroy the protective myelin coating around nerve fibres, leading to oligodendrocyte death and axonal damage [2, 3]. In multiple sclerosis lesions, activated microglia and macrophages are thought to contribute to neurodegeneration, as their number correlates with the extent of axonal damage [4]. Actually, activation of microglia/macrophages may represent one of the initial steps in demyelination in animal models of the pathology, preceding and possibly triggering T cell development and infiltration of blood-derived cells [4, 5]. Taking into account the role of microglia/macrophage activation in multiple sclerosis and several other neurodegenerative diseases, there is considerable interest in understanding the cellular pathways that underpin this phenotype. Development of new treatment strategies for such diseases would benefit from a deep knowledge of the metabolic basis of the inflammatory process. Following such motivation, we analyse here the changes in the lipidome associated with inflammation in mouse models of spinal cord injury by lysophosphatidylcholine (LPC).

To create the model, mice were injected into the spinal cord with a mixture of LPC species, which acts as a detergent, disrupting myelin and permeating cell’s membrane [6]. The injection produces a well-characterized demyelinating injury, consisting principally of macrophage/microglial infiltration and activation, reactive astrogliosis, perturbation of axonal homeostasis/axonal injury and oligodendrocyte progenitor cell proliferation and migration. The lesion predictably evolves over the period of a few weeks and is eventually capable of fully remyelinating [7]. The whole process is very similar to the inflammatory process in multiple sclerosis. Importantly, innate immune response mediated by microglia and infiltrating macrophages is pivotal during the regeneration process for cleaning myelin debris left by the damage. In addition, the proper degradation and turnover of the excess of myelin-derived lipids by phagocytes contribute to the proper resolution of inflammation [8]. As it can be seen, the whole process resembles that observed in multiple sclerosis and other inflammation-related diseases. Therefore, understanding lipid metabolism at cellular level in this demyelinating lesions, particularly in microglia and macrophages, will contribute to shed light on the metabolic basis of inflammation-related diseases.

Indeed, lipids are considered to play a relevant role in both initiation and resolution of inflammation [9]. However, getting further insights in the inflammation process faces the difficulty of the heterogeneity of the lesions. The traditional approach to study the lipidome of a tissue in the context of a pathology consists of making a sample homogenate, followed by an extraction and analysis by HPLC-MS. With such methodology, the spatial localization of the lipid species is lost, making difficult the interpretation of the results, as each cell population presents its own lipid fingerprint [10, 11], which evolves in a different way in the context of the pathology [12–15]. Lipid imaging mass spectrometry (LIMS) [16, 17] has completely changed this situation as it adds spatial localization to lipid composition, associating each cell type with its lipid fingerprint, provided that high-enough spatial resolution is reached [18, 19]. Such connection is of utmost importance to understand the role that lipids play in cell metabolism. Furthermore, given the intimate relationship between cell phenotype and lipid composition, LIMS is a kind of new molecular histology that enables visualization of the tissue from a metabolic point of view [20–22].

**Fig. 1:**
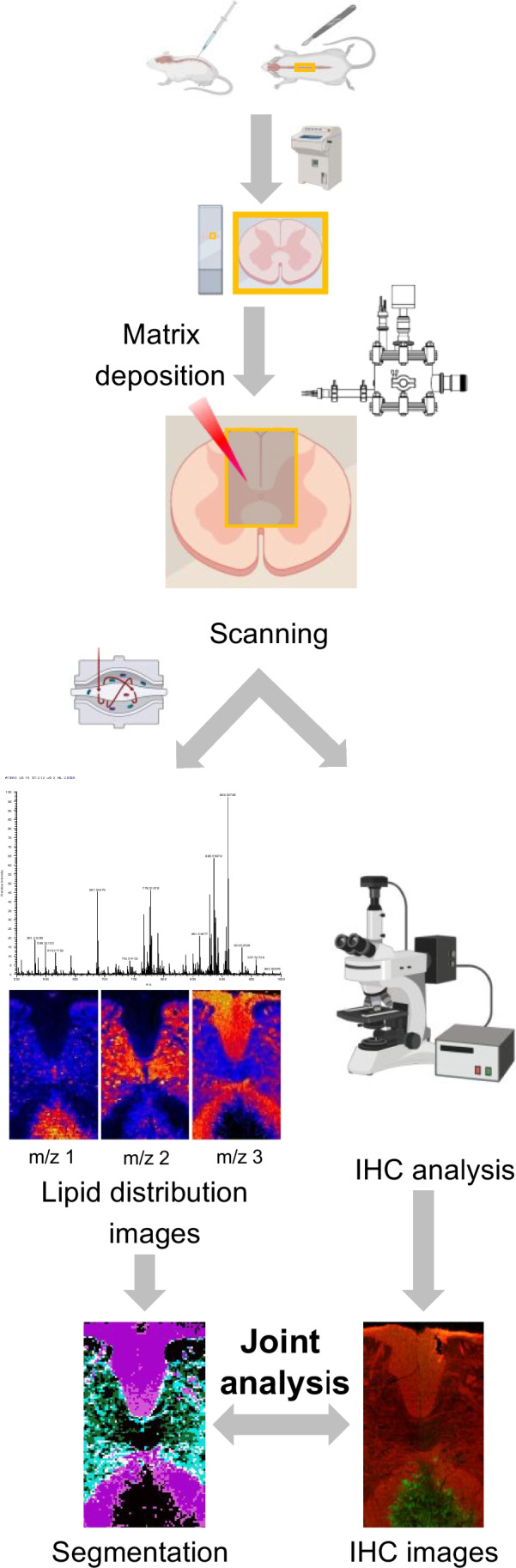
Workflow of the combined LIMS-IHC experiments. Mice were injected with LPC to create a lesion in the spinal cord. At 14 dpi, the animals were sacrificed, and the spinal cord was extracted and fixed in PFA 2% for 30 min before being deep frozen in liquid nitrogen. Sections obtained with the aid of a cryomicrotome were covered with matrix, explored in negative-ion mode using an Orbitrap mass spectrometer at 10 μm/pixel of spatial resolution. Then, the same sections were incubated with several antibodies labelled with fluorescent tags. Comparison between the segmentation images of the LIMS experiment and the IHC images helped identifying three areas: the lesion core, the peri-lesion and the uninvolved healthy tissue

Despite all these benefits, LIMS still has a long way ahead until fully unleashing its potential, mostly due to the lack of previous information on the lipid composition of the cells. Thus, it is necessary to combine it with well-established techniques, in order to understand the huge amount of information produced in each experiment [21]. Among the different strategies tested, microdissection, micro-extraction [23], correlation with RAMAN spectroscopy [24], microscopy [25] etc., combination with immunohistochemistry (IHC), either in the same sample or in consecutive sections, seems the most promising technique (Fig. 1) [21, 26, 27]. IHC is able to highlight the existence of cell populations or even sub-populations with different traits, such as proliferative potential in tumour cells, or the presence of tumour-associated fibroblasts [28–30]. The low damage induced in the samples by the laser during LIMS scanning makes possible to run IHC experiments on the same sample. Subsequent segmentation analysis of LIMS data and comparison with the IHC images may result in the correlation between lipid signatures and specific histologic areas or even cell populations [31, 32].

Several previous studies have dealt with the problem of characterizing the lipid signature of inflammation using LIMS, mostly by integrating the signal from the whole lesion without taking into account its heterogeneity [33–40], or restricting the analysis to two regions of interest (ROI): normal and damaged tissue [24, 41] and comparing them with the optical image. An attempt to associate lipid signatures to infiltrating cells by correlating LIMS and IHC images was recently published by Quainco et al. [26], but the moderate spatial resolution of the LIMS images precluded a precise assignment.

Here, we use LIMS at 10 μm/pixel of spatial resolution to record images of lipid distribution over sections of mouse spinal cord with LPC-induced demyelinated lesions. The same sections were afterwards used to run IHC experiments (Fig. 1). Comparison between the segmentation images of the LIMS experiments and those from IHC allowed us to extract lipid fingerprints of the lesion core, the microglia and macrophage populations accumulating in the periphery of the lesion (the peri-lesion) and the uninvolved white matter. As it will be demonstrated, the three areas present well-defined, differentiated and reproducible lipid fingerprints, enabling their classification using several algorithms.

## Methods

### Animals

All experiments were performed in C57BL/6 mice (Janvier Labs) according to the procedures approved by the Ethics Committee of the University of the Basque Country (UPV/EHU), M20/2019/021. Animals were handled in accordance with the European Community’s Council Directive. Animals were kept under conventional housing conditions (22 ± 2 °C, 55 ± 10% humidity, 12-h day/night cycle and with ad libitum access to food and water) at the University of the Basque Country animal unit. All possible efforts were made to minimize animal suffering and the number of animals used.

### LPC-induced demyelination

To analyse remyelination in mice, we performed LPC-induced demyelination in the spinal cord in nine 14-week-old male mice. The lesions were induced by stereotaxic injection of 0.5 μL of 1% LPC (Sigma) in saline solution, as previously described [42]. Briefly, animals were anesthetized by intraperitoneal injection of a solution of ketamine (100 mg/kg, Sigma) and xylazine (10 mg/kg, Sigma). The tissue covering the vertebral column was removed by two longitudinal incisions into the *longissimus dorsi*, and the intravertebral space of the 13th thoracic vertebra was exposed by removing the connective tissue, after fixing the animal in the stereotaxic frame. Dura mater was then pierced using a 30G needle, and LPC was injected via a Hamilton syringe attached to a glass micropipette, using a stereotaxic micromanipulator.

The lesion-specific site was marked with sterile charcoal, so that the area of tissue at the centre of the lesions could be unambiguously identified afterwards. Following LPC injection, the wound was sutured, and mice were allowed to recover. Buprenorphine (0.1 mg/kg) was administered by subcutaneous injection as postoperative analgesic treatment. To assess the response to demyelination, 14 days post injection (dpi), the animals were euthanized, perfused with 2% PFA for 15–20 min, and the spinal cords were post-fixed in 2% PFA for another 30 min. Then, the samples were immersed in liquid nitrogen and stored at − 80ºC.

### LIMS experiments

In order to evaluate the changes in the lipid signatures in the context of LPC-induced demyelination, 12-µm-thick coronal sections were obtained from the mice spinal cords at 14 dpi, with the aid of a cryomicrotome. The sections were covered with 1,5-diaminonaphthalene, using an in-house designed sublimator [43], and introduced into the modified [44] MALDI source of a MALDI-LTQ-Orbitrap XL (Thermo Fisher, San Jose, CA) available in the Analytical Services of the University of the Basque Country (UPV/EHU). The following parameters were used during the sublimation process: matrix temperature 393 K; sublimation time 6 min; and sample temperature 278 K. During the whole process, the vacuum was maintained at < 0.1 mbar. Data acquisition was performed at 10 µm/pixel, using two microscans of 10 shots/each, using a laser energy of ~ 10–20 μJ/pulse and a mass resolution of 60,000 at m/z = 400. All experiments were recorded in negative-ion mode using the Orbitrap analyser. The observation window was set to 550–1000 Da, where most glycerophospholipid and sphingolipid classes appear. Negative polarity was chosen as it enables the detection of species from a larger number of lipid classes, and the spectra are easily to annotate, due to the presence of a reduced number of adducts [11, 45, 46]. Among the large number of matrices available to work in this polarity [47, 48], we chose DAN, as its deposition by sublimation results in uniform films of small crystals, and it has demonstrated to produce excellent signal intensity with a low background [49].

The spectra obtained in this way were processed using our own software developed in MATLAB (MathWorks, Nantick). Briefly, the spectra were aligned, normalized using the total ion current and summed to obtain an average spectrum that was used to extract the peaks with intensity above 0.5% of the strongest peak. The list of peaks created that way was used to extract the m/z from the spectra at each pixel, achieving in this way a substantial reduction in the data size and leaving the data ready for subsequent analysis. Then, the images were segmented using the lipid fingerprint at each pixel and a divisive hierarchical clustering-rank compete (DHC-RC) algorithm [50]. Briefly, it is a variation of the hierarchical divisible analysis algorithm [51] that uses a variable number of random walkers to divide the image according to the correlation between the pixels. Capturing all the details of the images required in some cases to isolate a given segment and re-analyse it. As the algorithm uses the correlation of lipid fingerprint between pixels, the strong difference between, for example, lesion and healthy tissue hampers otherwise detecting the smaller correlation differences between other areas.

Annotation of the lipids was carried out by comparison of the m/z value with those in lipid maps and the MS/MS spectra of those peaks with enough intensity to record such data (see supplemental figures S4-S24).

### Immunohistochemistry

After exploration in the mass spectrometer, the spinal cord sections were analysed by IHC (Fig. 1), following the protocol in ref [21]. First, the sections were fixed with 4% paraformaldehyde in phosphate buffered saline (Sigma-Aldrich, MO, USA) for 10 min at room temperature. Then, the matrix remaining after MALDI scanning was removed by washing the sections during 5 min using a cold methanol/acetone (1:1) mixture (minimum purity 99.5%, Scharlab, Barcelona, Spain). Tissue sections were permeabilized, and non-specific labelling was blocked using 4% of serum of the species in which secondary antibody was obtained in 0.1 M PBS and 0.1% Triton X-100 for 1 h at room temperature. Next, samples were incubated with primary antibodies overnight at 4 °C in 4% serum and 0.1% Triton X-100. After washing with PBS, corresponding fluorochrome-conjugated antibodies Alexa Fluor 488 or 594 conjugated goat secondary antibodies (1:250; Invitrogen) and Hoechst 33258 (1.5 μg/ml; Sigma-Aldrich) for nuclei labelling were applied, and samples were incubated during 1 h at room temperature. After washing, they were mounted using Glicergel mounting medium (Dako). Primary antibodies used for immunofluorescence (IF) on these tissues included mouse anti-myelin basic protein (MBP, 1:1000; #808401 BioLegend), rabbit anti-Iba1 (1:500; #019-19741 Wako Chemicals) and mouse anti-inducible nitric oxide synthase (iNOS, 1:100; # 610329 BD Biosciences).

Images were acquired using either a Leica TCS STED SP8 or a Zeiss LSM800 confocal microscope with the same settings for all samples. All the fluorescence image analysis was performed with the ImageJ software (National Institutes of Health; NIH).

## Results and discussion

### Comparison between LIMS segmentation images and IHC fluorescence images

**Fig. 2:**
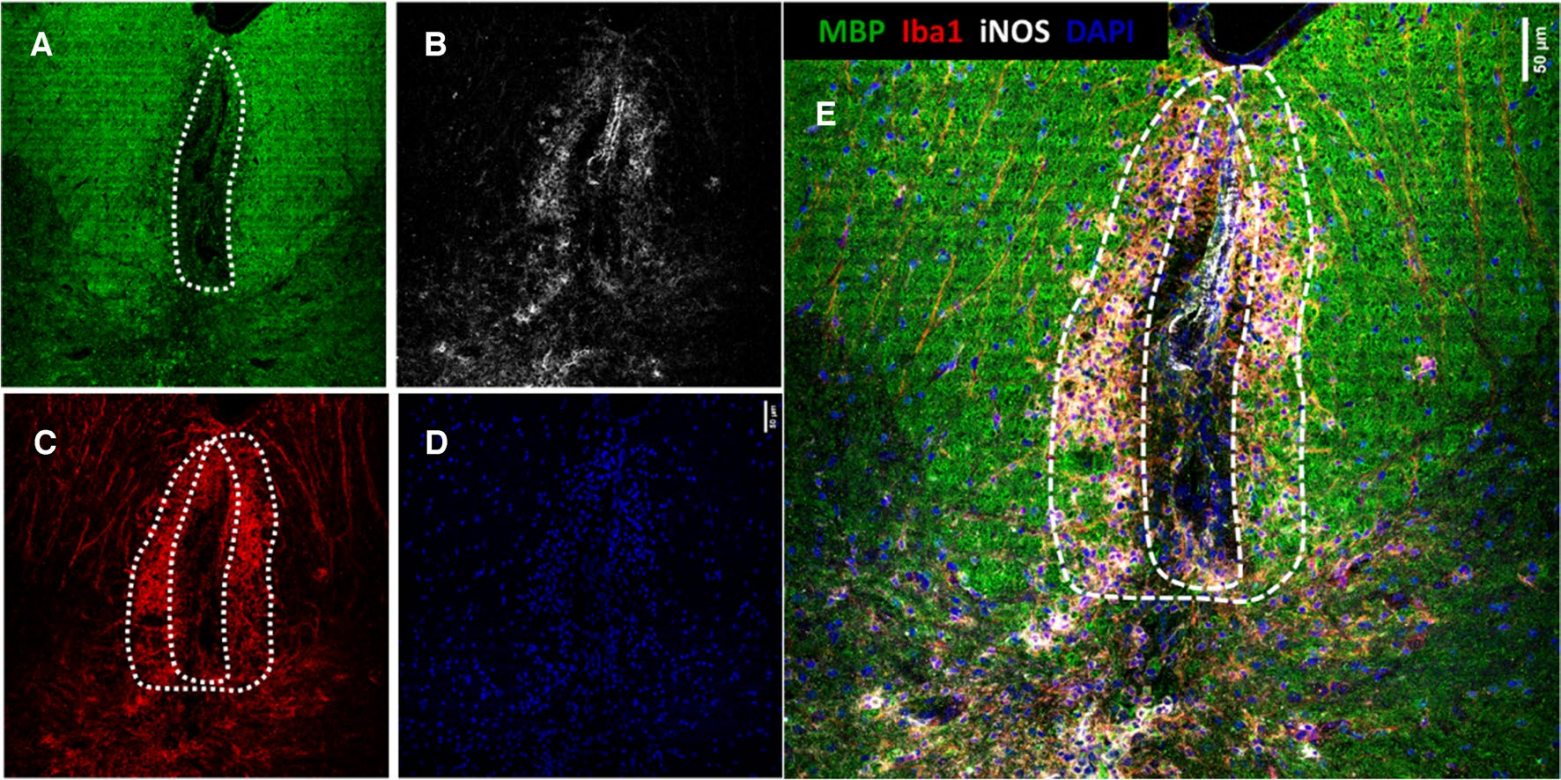
Immunofluorescence (IF) images of a coronal section of mouse spinal cord at 14 dpi with LPC. **A** MBP staining, highlighting the tissue architecture; **B** iNOS staining highlights the proinflammatory microglia/macrophages in the lesion and the vascular tissue; **C** Iba1 marks the microglia inside the lesion; **D** DAPI stains cell nuclei; **E** merge of the four images, showing a clear co-localization of the reactive glia and the margin of the lesion. Scale bar 50 μm

Injection with LPC produced massive demyelination, inflammation and axonal damage. However, at 14 dpi, the regeneration response has already started. Oligodendrocyte progenitors have migrated to the lesion, and they have differentiated into mature oligodendrocytes in order to remyelinate the lesion [6]. Typical LPC-induced demyelinated lesions are illustrated in Fig. 2, where an example of fluorescence image of IHC recorded over a section of injured mouse spinal cord is shown. MBP staining (green, Fig. 2A) enables visualizing the demyelinated area as well as the tissue architecture (white and grey matter), as it is a marker of myelin. Different green tones delimit the lesion (dark green in the centre of the image), the unaffected white matter (bright green at both sides of the lesion) and the grey matter (darker green in the lower part of Fig. 2A). On the other hand, the extension of the lesion is clearly delimited by the combination of iNOS (panel B) and Iba1 (panel C). Iba1 staining enables visualization of microglia accumulating in the core as well as in the margins of the lesions, whereas iNOS highlights the presence of proinflammatory or activated microglia, preferentially in the margins of the lesions. Finally, DAPI, a common marker of cell nuclei, allows one to visualize the accumulation of inflammatory and infiltrating cells in the lesions. The combination of all four markers (Fig. 2E) shows that the treatment with LPC caused massive demyelination in the place of the injection, as well as an inflammatory reaction extending at both sides from the focal injection point. It also highlights the heterogeneity of the lesion. Based on the analysis of the IHC images, we defined three regions, which match with previous descriptions of this type of injuries [6]: the lesion centre or lesion core, characterized by a massive demyelination; the peri-lesion, characterized by the presence of activated iNOS^+^ microglia and an active processes of demyelination; and the uninvolved white matter. We thereafter refer to the two regions conforming the lesion as lesion core and peri-lesion, respectively.

**Fig. 3:**
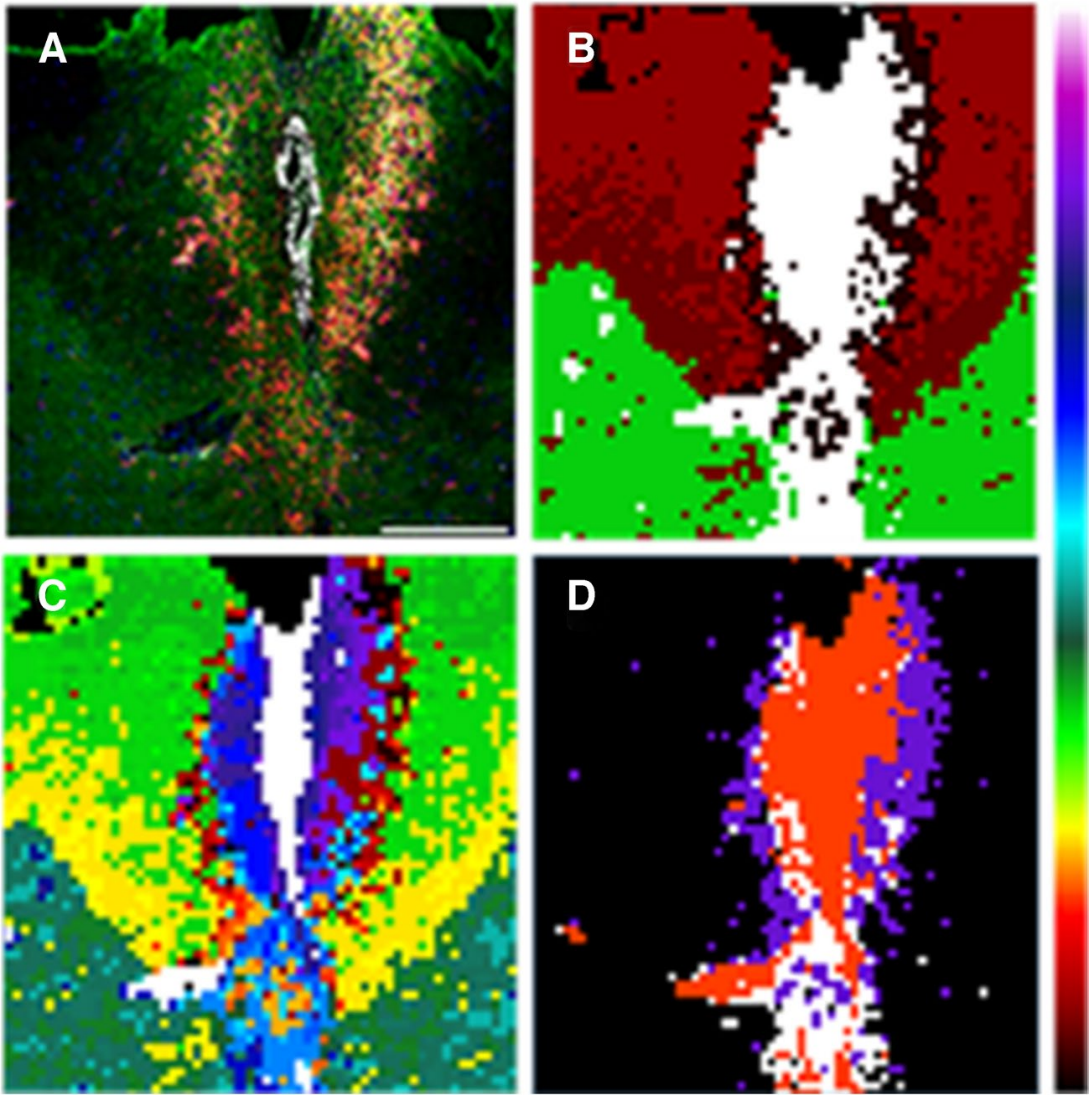
Comparison between (**A**) the IHC image and (**B**–**D**) the segmentation images of the corresponding LIMS experiment carried out over the same section of mouse spinal cord 14 dpi. With five segments (**B**), it is already possible to distinguish the lesion (white), the peripheral of the lesion (black) and the uninvolved grey (red) and white matter (green). Increasing the number of segments (**C**), it is possible to better separate peri-lesion and lesion core, but those areas also appear divided between several segments. Black and white segments in (**B**) were segregated and re-segmented into four segments (**D**). Now the lesion’s pixels are grouped in the red cluster, while the peri-lesion corresponds to the purple pixels. The segments were coloured following the colour bar in the figure, using the correlation between lipid fingerprints: those segments with colours closer in the bar present more similar lipid fingerprints. Scale bar 200 μm

Figure 3 shows a comparison between the IHC fluorescence image of one of the sections and the segmentation images of the corresponding LIMS experiment recorded over the same section. Using our segmentation software, it is possible to group the pixels in the LIMS experiment attending to their lipid fingerprint. Furthermore, the colour of each segment is assigned using the colour bar in Fig. 3 and the correlation, so the two segments with the lowest correlation receive the colours at the two ends of the scale (white and black). For the rest of the segments, the proximity of their colours in the scale indicate the similarity of their lipid fingerprint. When the number of segments in the experiment of Fig. 3 is set to five, the area occupied by the lesion core is immediately segregated and appears in a single white segment. There is also a clear division between grey matter (green segment) and the uninvolved white matter, which appears in two segments with different red tones. Interestingly, the margins of the lesion appear in the black segment.

Although the image in Fig. 3B is a good starting point for the analysis of the lipid signatures of spinal cord injury, it does not reflect the complexity of the IHC image. Especially important is to be able to extract the lipidome of the microglia/macrophage cells, which form the white–red bands in Fig. 3A and that define the peri-lesion. Those areas are only segregated when the number of segments is increased beyond a reasonable point (Fig. 3C). This is a well-known problem of the hierarchical algorithms: sometimes they take erroneous bifurcations, dividing the data into meaningless segments [52].

A way to circumvent this problem is to segregate the segments that match with the desired histologic area and re-analyse them. Such strategy allowed us to unravel in previous works the architecture of complex tissues [21]. Thus, the white and black segments in Fig. 3B were segregated and re-analysed, setting the number of segments to four. The resulting image (Fig. 3D) shows that the pixels defining the margin of the lesion were grouped into a single purple segment that matches the area defined as peri-lesion in the IHC fluorescence image, and the rest of the lesion (the lesion core) appears as white and red segments. In this way, it was possible to isolate the lipid fingerprint of the area containing the phagocytic microglia cells in the peri-lesion.

Similar procedure was followed with the rest of the samples (Figure S1): initial segmentation of the LIMS experiment, extraction of the segment defining the lesion by comparison with the IHC images and re-segmentation to segregate the different areas of the lesion, in those cases in which the lesion was not properly defined with the initial segmentation. As it can be seen in the figure, lesions are highly heterogeneous. Despite the care taken in producing similar lesions, both the size and the position changed between animals and only through comparison with the IHC images it was possible to correctly identify the areas with mainly inflammation (lesion core), the border of the lesion (peri-lesion), where the infiltrating cells are located, and the unaffected areas.

Once the areas corresponding to lesion core, peri-lesion and healthy white matter were correctly identified, it was possible to compare the lipid expression in all three areas. Figure 4 compares the relative abundance of the lipid species identified, grouped into classes. Comparison of the relative abundance of lipid classes may be found in Figure S2, while some MS/MS spectra may be found in supplemental Figures S4-S24. Fragmentation spectra of sulfatides were omitted as they only serve to confirm the lipid class, but they usually do not present fragments that enable precise assignment of the fatty acids.

**Fig. 4:**
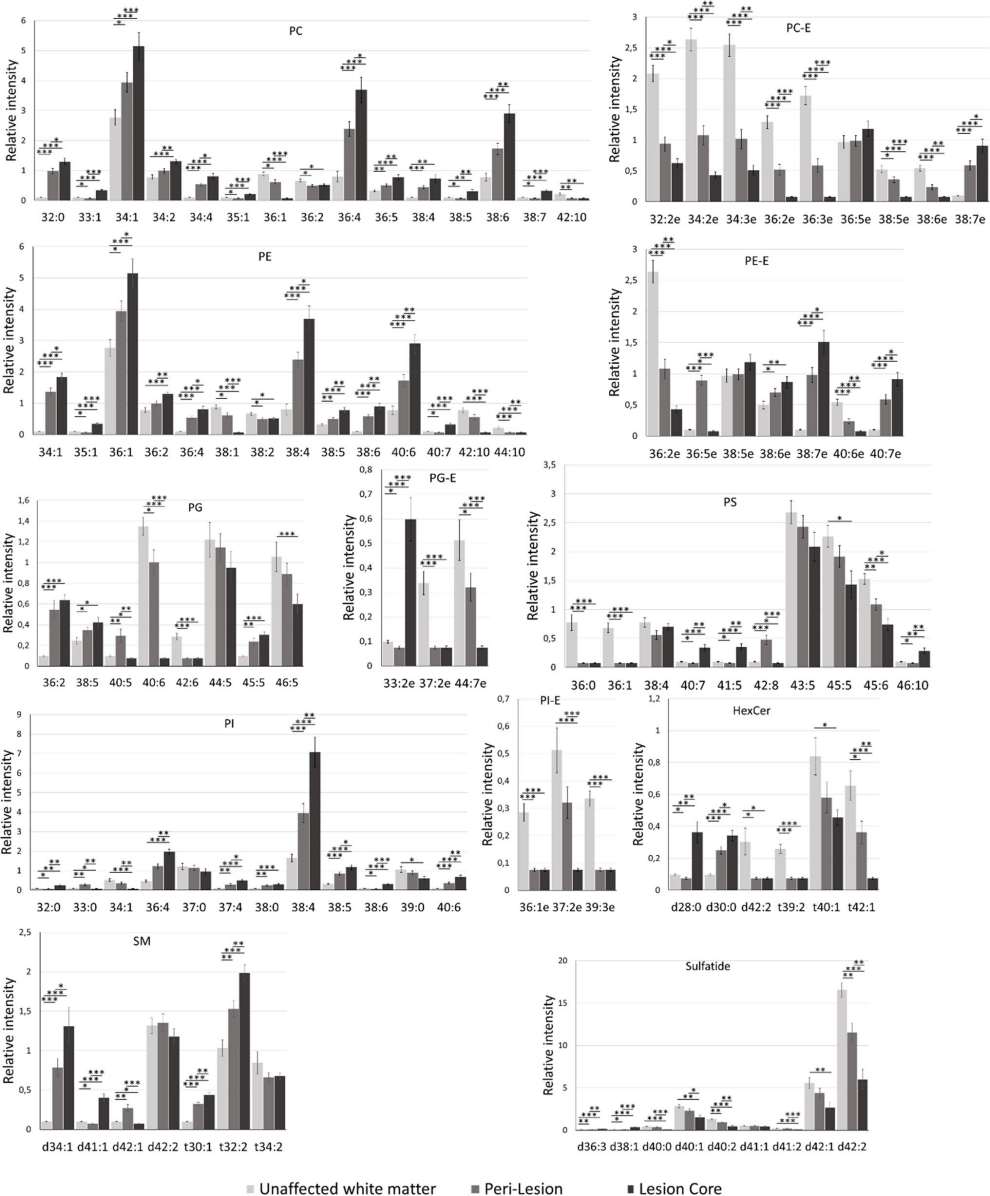
Changes in the relative abundance of the identified lipid species, between unaffected white matter, peri-lesion and lesion core. The asterisks correspond to the significance of the changes observed: * *p* < 0.05; ** *p* < 0.01; *** *p* < 0.0001 in a *t*-test

A fast look at the figure shows a clear increase in the total amount of PC and PE from healthy tissue to peri-lesion and lesion core. Such increase is mirrored by a general decrease in PC-E species. Meanwhile, PE-E species follow a different trend, with a strong decrease in PE 36:2e and 40:6e and an increase in 38:6e, 38:7e and 40:7e. PE 36:5e is the only species whose abundance is larger in the peri-lesion.

PI is usually more abundant in grey matter and is specially localized in neurons [53]. In this case, there is a strong increase in PI 38:4, the main store of arachidonic acid (AA, 20:4) and other species that may also contain this fatty acid: 36:4, 37:4, 38:5, 38:6 and 40:6, although the latter could also be a different isomer (PI 18:0/22:6). AA is usually involved in inflammatory processes, as it is used to produce prostaglandins [54].

A general decrease in the relative abundance of the PS species was also observed from unaffected white matter to inflamed regions and to lesion core. Nevertheless, the annotations of PS must be taken with caution, as the fragmentation of PI species overlaps with the PS m/z. Regarding phosphatidylglycerol (PG), there seems to be remodelling of the fatty acid composition. This glycerophospholipid is related with the mitochondrial activity, as it is the main building block for the production of cardiolipins. Its change in the context of inflammation may be, therefore, connected to the modification of the mitochondrial activity.

Regarding the sphingolipids, there is a clear increase in the relative abundance of SM and a decrease in sulfatides. The latter is almost exclusively found in oligodendrocytes [53]. Its disappearance may be due to the tissue degradation produced by the demyelination process. Importantly, myelin-derived sulfatides are endogenous ligands for TLR4 receptor and modulate innate immune activation. In contrast to sulfatides and galactosylceramide, that are almost exclusively located in myelin, SM is present in all the membranes. Therefore, the increase in SM could be related to microglia activation and proliferation, more than to demyelination.

**Fig. 5:**
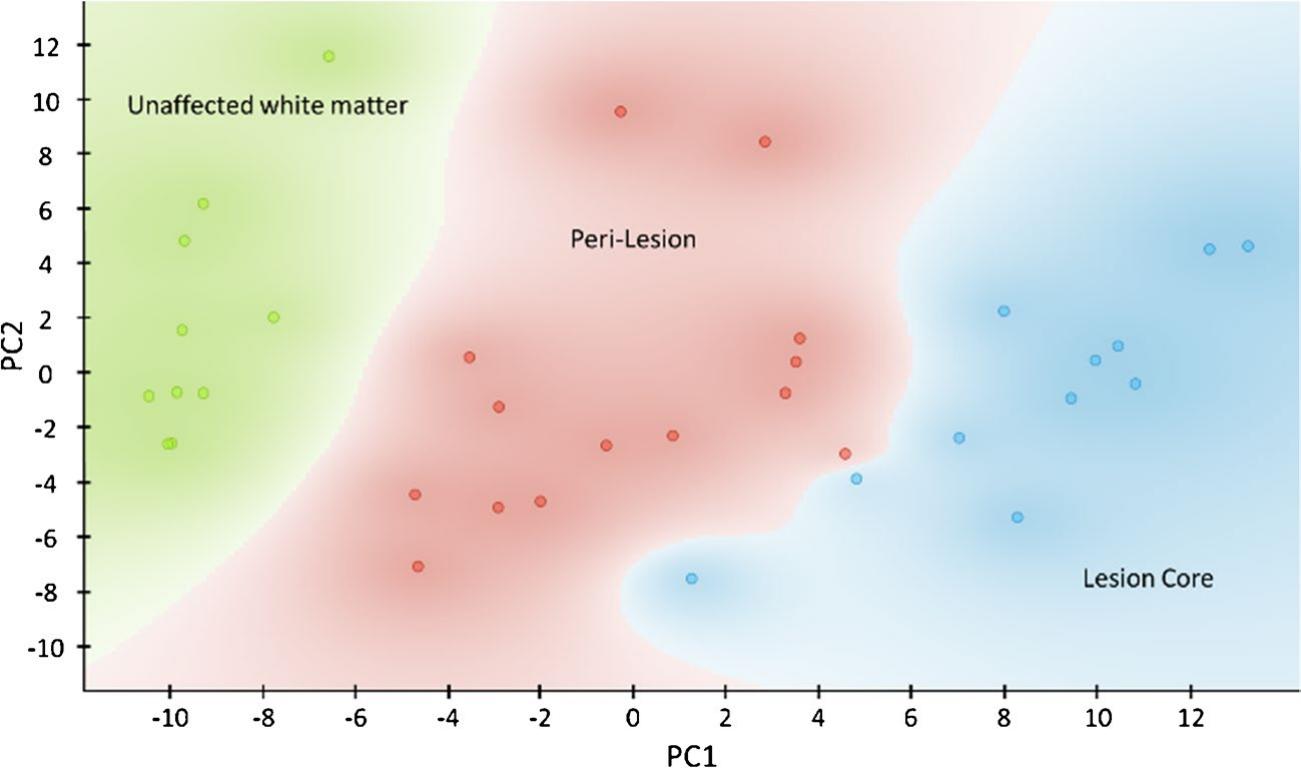
Score plot of a PCA analysis of the lipid fingerprints of unaffected white matter (green), inflamed tissue (red) and lesion (blue), obtained from the samples analysed in this work. The area occupied by each type of sample was automatically coloured by the software, taking into account the identity of the nearest samples. PC1 explains 46% of the variance and PC2 explains 15%

The robustness of the changes in the relative abundance of lipid species reported in Fig. 4 is certified by the neat separation between the lipid fingerprints of the three areas, obtained in a simple PCA analysis, reported in Fig. 5. As it can be seen, the lipid signatures from each type of tissue are grouped together, and there seems to form a gradient along PC1, from unaffected tissue to the peri-lesion (the margins of the lesion) and to the lesion core itself, where demyelination is complete. Using the lipid species reported in Fig. 4, it is possible to set up models to classify each type of tissue (Table S1 and Figure S3 of the supplemental information). Among the algorithms tested, random forest was able to classify the lipid signatures from the three types of tissue with 100% precision, specificity and sensibility, demonstrating that the lipid fingerprint of each area was correctly isolated with the method employed and that, despite the heterogeneity of the lesions, the changes in lipid expression that accompanied the demyelination process are preserved among the individuals examined. In other words, there is a lipid signature of the demyelination process. Moreover, lipid signature in the peri-lesion, characterized by the existence of an active process of demyelination, is positioned between normal withe matter and lesion core. Therefore, the lipid signature will mainly represent the process of demyelination more than the inflammatory reaction around the lesion. However, small and specific changes observed in the peri-lesion, and not in the lesion core, could help to shed light into the lipidic changes associated specifically to the microglia activation during demyelination.

## Conclusions

We presented here a combination of LIMS with IHC to examine and isolate the lipid fingerprint of unaffected white matter tissue, the margin of the lesion and the epicentre of the lesion in animal models of demyelination. Both sets of experiments were carried out over the same sections, in order to achieve an optimum match between LIMS segmentation images and IHC fluorescence images. In this way, despite the variety of geometries of the lesions, it was possible to identify each type of tissue in every sample. The latter was demonstrated by PCA analysis, in which a perfect separation along PC1 was obtained. Classification models based on random forest algorithm also enabled a perfect classification of each type of tissue, validating the robustness of the protocol employed. Among the changes observed, there was a profound alteration of all lipid classes examined. The consistence of such changes among individuals points to the existence of a lipid signature of the demyelination process. These results set the foundations to examine the changes in the metabolic bases of the inflammation process associated to different pathologies.

### Supplementary Information

Below is the link to the electronic supplementary material.

Supplementary file1 (PDF 2614 KB)
