# Supplementary material for: Combining imaging mass spectrometry and immunohistochemistry to analyse the lipidome of spinal cord inflammation
Source: Anal Bioanal Chem. 2024 Feb 7;416(8):1923–33. doi: 10.1007/s00216-024-05190-3 (PMC10902057; doi:10.1007/s00216-024-05190-3)
Supplement: Supplementary file 1 — Supplementary file1 (PDF 2614 KB) [file 216_2024_5190_MOESM1_ESM.pdf]

Supporting Information for:

Combining Imaging Mass Spectrometry and Immunohistochemistry to Analyse the Lipidome of Spinal Cord Inflammation

Ibai Calvo,±[a] Alejandro Montilla, ±[b,c] Cristina Huergo,[a] Lucía Martín-Saiz,[a] Javier Martín-Allende,[d] Vanja Tepavcevic [b], María Domercq*[b,c] and José A. Fernández* [a]

[a]*Dep. of Physical Chemistry, Fac. of Science and Tecnology, University of the Basque Country (UPV/EHU). Bº Sarriena s/n, Leioa 48940, Spain.*
[b] *Achucarro Basque Center for Neurosciencie. Bº Sarriena s/n, Leioa 48940, Spain.*
[c]*Dep Neuroscience, Fac. of Medicine, University of the Basque Country (UPV/EHU). Bº Sarriena s/n, Leioa 48940, Spain.*
[d]*Dep. of Languages and Computer Systems, School of Engineering University of the Basque Country (UPV/EHU), Paseo Rafael Moreno "Pitxitxi", n. 2/3 48013 - Bilbao, Spain.*
[±]These two authors contributed equally to this work.

Corresponding authors:

María Domercq
maria.domercq@ehu.eus
Phone +34 946 015 717

José A. Fernández
email: josea.fernandez@ehu.eus
Phone: +34 94 601 5387
Fax: +34 94 601 3500

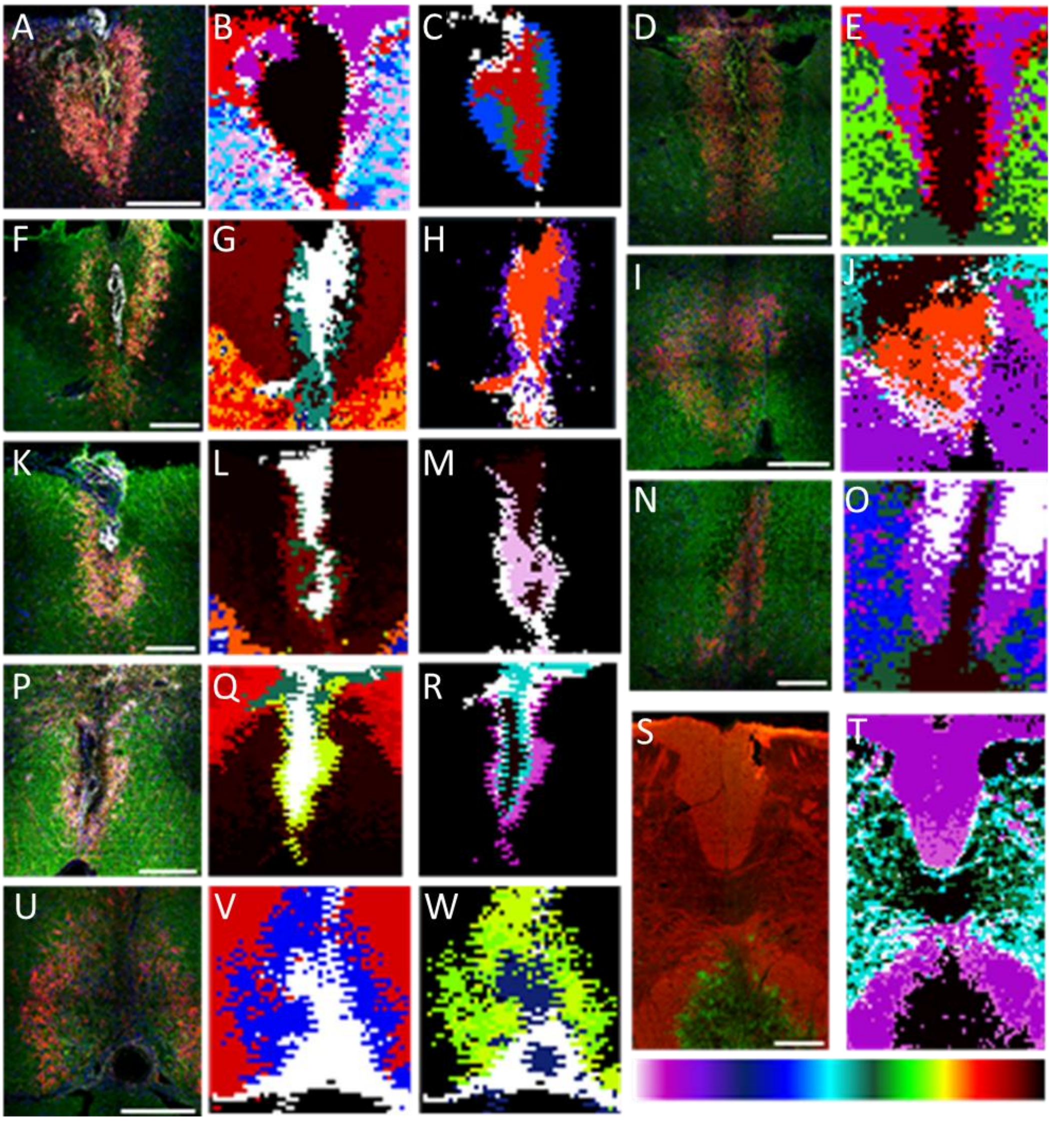

Figure S1.Comparison between the IHC images (**A, D, F I, K, N, P, S** and **U**) and the segmentation images of the corresponding LIMS experiment, obtained setting the number of segments to between 5 and 10 (**B, E, G, J, L, O, Q, T** and **V**). In those cases in which the initial segmentation was not able to describe adequately the lesion, the segment(s) containing it was segregated and re-analysed (**C, H, M, R**, and **W**). IHC in S has the colours reversed: Iba1 in green and MBP in red. Segment colours were assigned from the colour bar in the image, according to the correlation between them. All LIMS experiments carried out in negative-ion mode at 10 μm/pixel. Scale bar: 200 μm

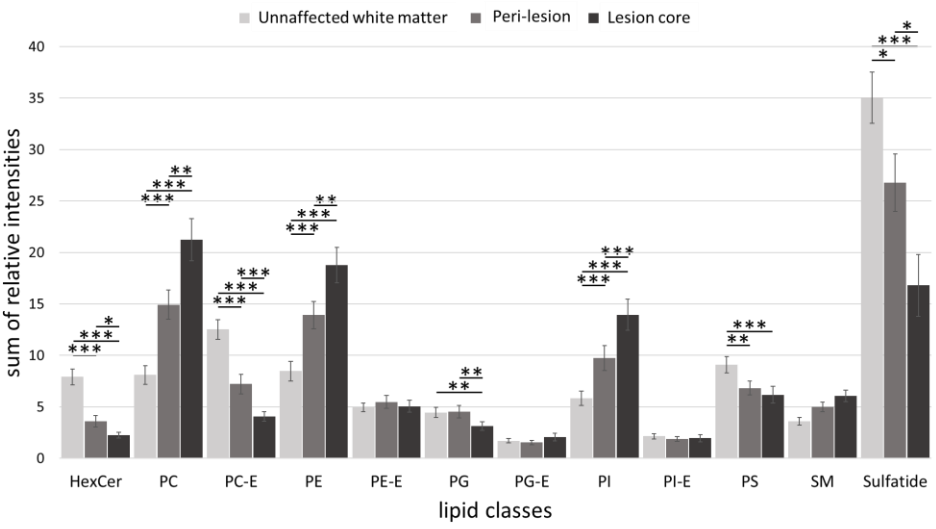

Figure S2. Relative abundance of the lipid classes studied in this work in the three areas identified in the samples. The asterisks correspond to the significance of the changes observed: *: $p < 0.05$; **: $p < 0.01$; ***: $p<0.0001$ in a T-test.

Model	AUC	CA	F1	Precision	Recall
SVM	0.999	0.971	0.971	0.973	0.971
Random Forest	1.000	1.000	1.000	1.000	1.000
Naive Bayes	0.993	0.914	0.913	0.914	0.914
Logistic Regression	1.000	1.000	1.000	1.000	1.000

Table S1. Performance of the four models tested to classify the lipid signatures extracted from the sections. All models exhibit outstanding performance but in the case of those based on Random Forest and Logistic regression algorithms the classification is perfect, with absence of false positive and false negative cases.

A.

Predicted

Actual	Lesion Core	Peri-lesion	Unaffected white matter	Σ
Lesion Core	11	0	0	11
Peri-lesion	0	14	0	14
Unaffected white matter	0	1	9	10
Σ	11	15	9	35

B.

Predicted

Actual	Lesion Core	Peri-lesion	Unaffected white matter	Σ
Lesion Core	11	0	0	11
Peri-lesion	0	14	0	14
Unaffected white matter	0	0	10	10
Σ	11	14	10	35

C.

Predicted

Actual	Lesion Core	Peri-lesion	Unaffected white matter	Σ
Lesion Core	11	0	0	11
Peri-lesion	1	12	1	14
Unaffected white matter	0	1	9	10
Σ	12	13	10	35

D.

Predicted

Actual	Lesion Core	Peri-lesion	Unaffected white matter	Σ
Lesion Core	11	0	0	11
Peri-lesion	0	14	0	14
Unaffected white matter	0	0	10	10
Σ	11	14	10	35

Figure S3. Confusion matrices for the four classification models tested. **A)** support vector machine (SVM); **B)** Random Forest; **C)** Naïve Bayes; **D)** Logistic Regression. Perfect classification of the samples was achieved with Random Forest and Logistic Regression.

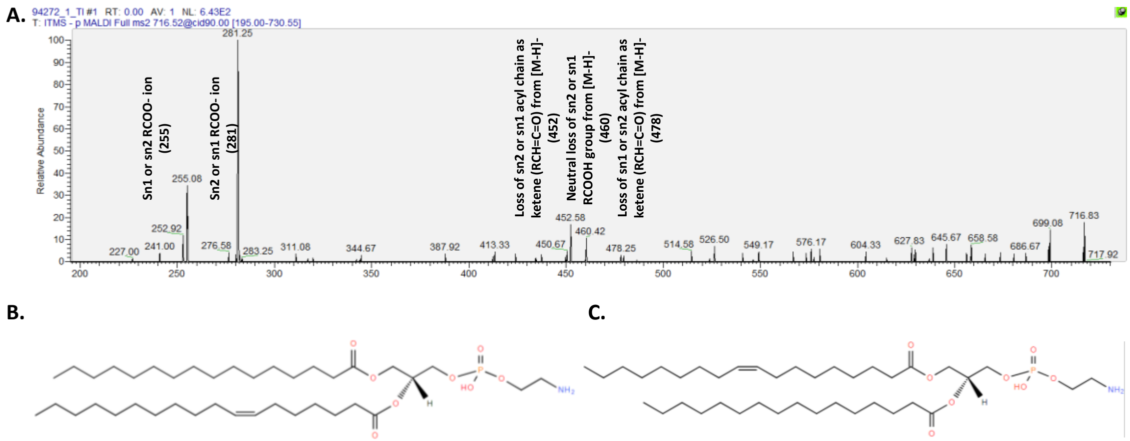

Figure S4. A) Fragmentation spectrum of m/z = 716.524 in negative polarity using a laser energy of 40 µJ. The fragments point to **B)** PE (16:0_18:1) or **C)** PE (18:1_16:0). Position of the double bonds was not determined.

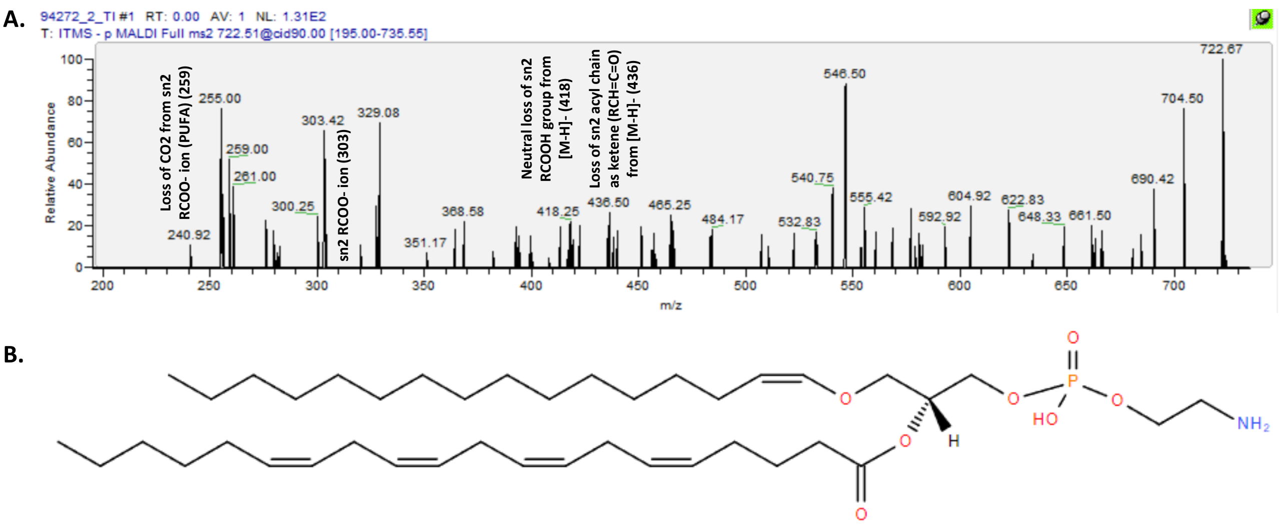

Figure S5. A) Fragmentation spectrum of m/z = 722.513 in negative polarity using a laser energy of 40 µJ. The fragments point to **B)** PE P-16:0/20:4. Position of the double bonds was not determined.

A.

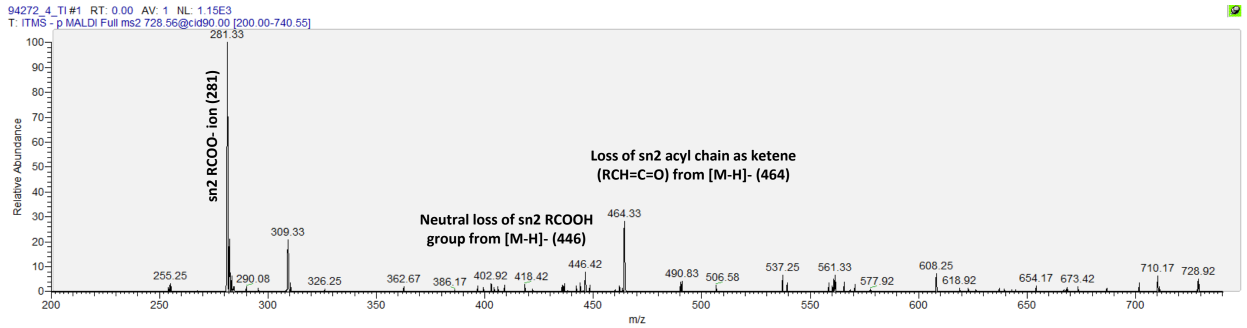

B.

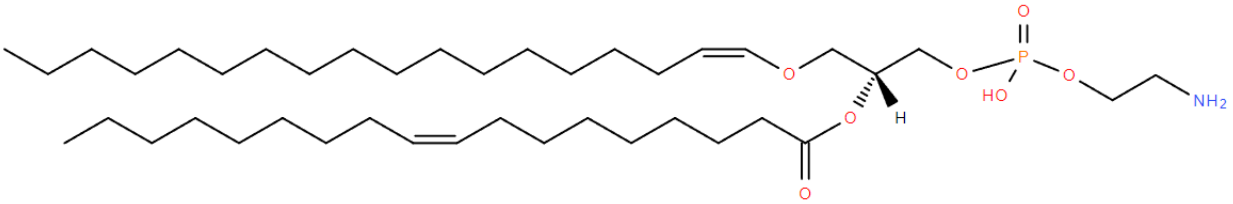

Figure S6. A) Fragmentation spectrum of m/z = 728.560 in negative polarity using a laser energy of 40 µJ. The fragments point to **B)** PE P-18:0/18:1. Position of the double bonds was not determined.

A.

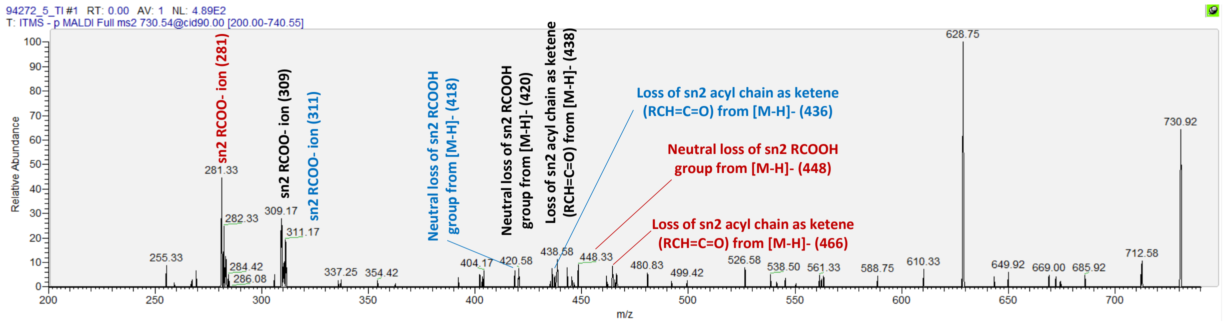

B.

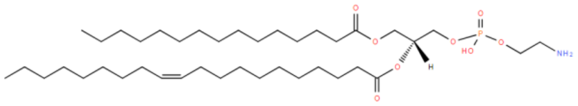

C.

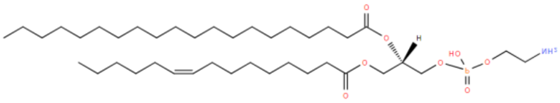

D.

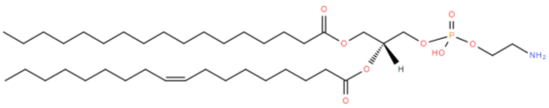

Figure S7. A) Fragmentation spectrum of m/z = 730.538 in negative polarity using a laser energy of 40 µJ. The fragments point to **B)** PE 15:0_20:1 and **C)** PE 15:1_20:0 (blue labels) and **D)** PE 17:0/18:1 (red labels). Position of the double bonds was not determined.

A.

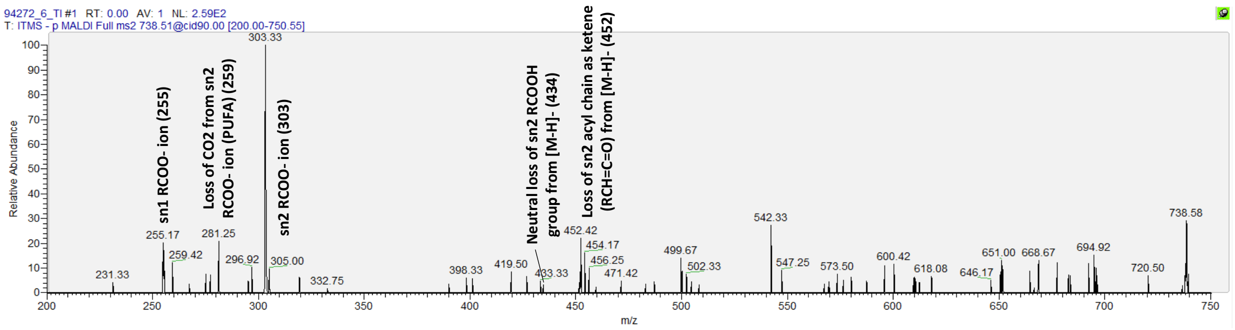

B.

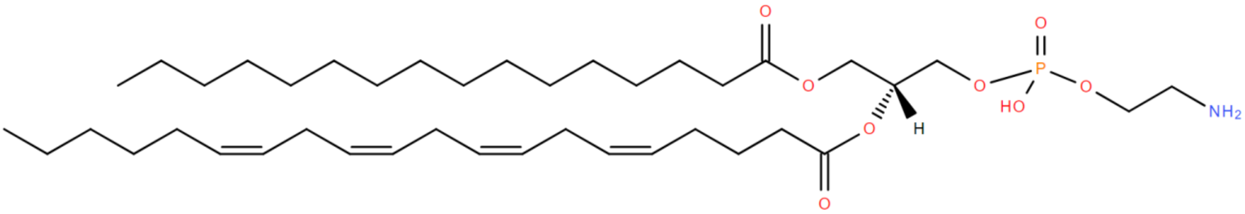

Figure S8. A) Fragmentation spectrum of m/z = 738.508 in negative polarity using a laser energy of 40 µJ. The fragments point to **B)** PE 16:0/20:4. Position of the double bonds was not determined.

A.

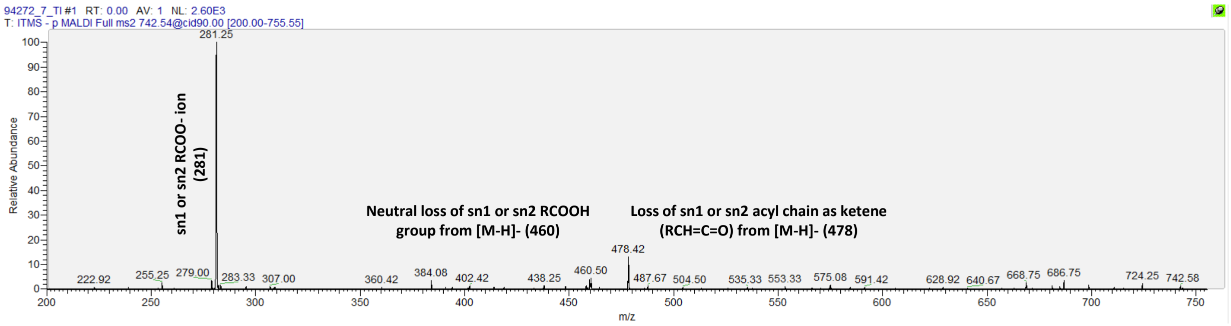

B.

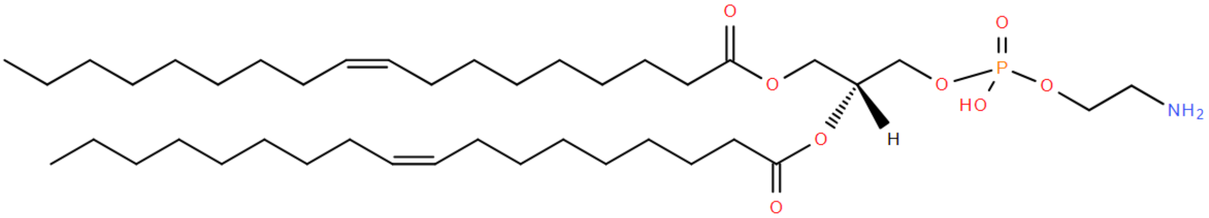

Figure S9. A) Fragmentation spectrum of m/z = 742.542 in negative polarity using a laser energy of 40 µJ. The fragments point to **B)** PE 18:1/18:1. Position of the double bonds was not determined.

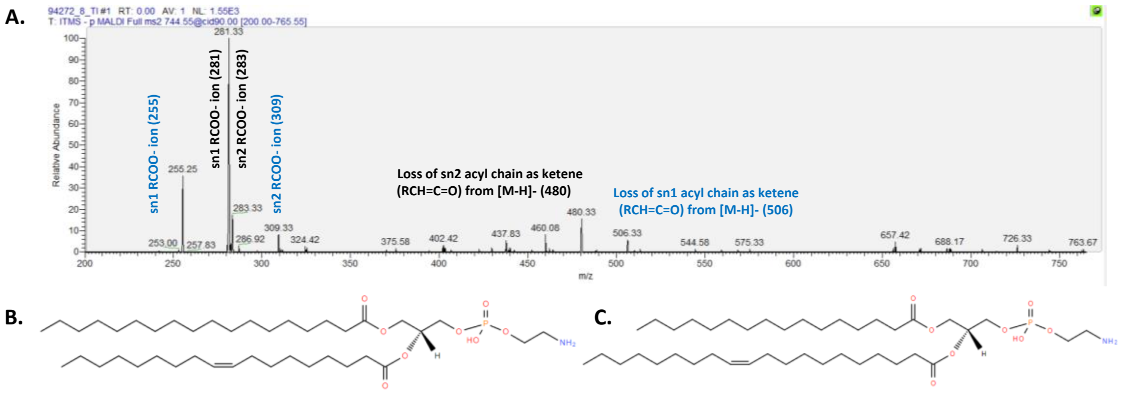

Figure S10. A) Fragmentation spectrum of m/z = 744.544 in negative polarity using a laser energy of 40 µJ. The fragments point to **B)** PE 18:1_18:0 or PE 18:0_18:1 and **C)** PE 16:0_20:1 (blue). Position of the double bonds was not determined.

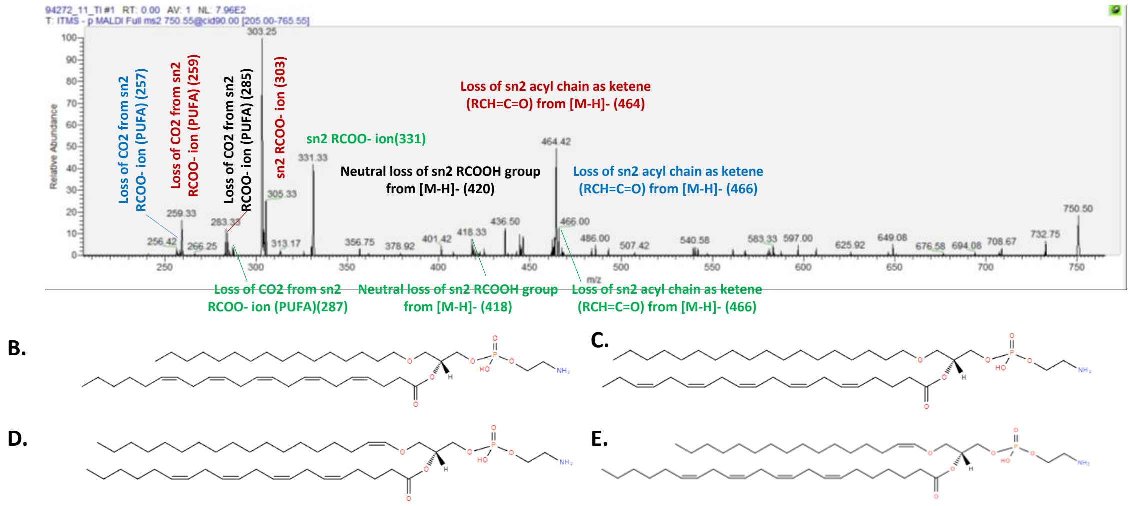

Figure S11. A) Fragmentation spectrum of m/z = 750.548 in negative polarity using a laser energy of 40 µJ. The fragments point to contribution from four different species: **B)** PE O-16:0/22:5, **C)** PE O-18:0/20:5 (blue), **D)** PE P-18:0/20:4 (red) and **E)** PE P-16:0/22:4 (green). Position of the double bonds was not determined.

A.

B.

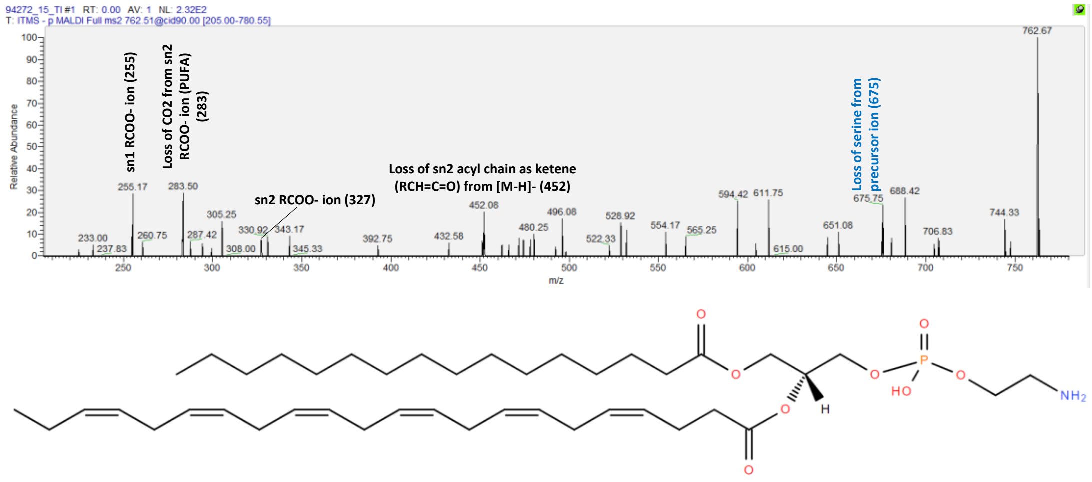

Figure S12. **A)** Fragmentation spectrum of m/z = 762.507 in negative polarity using a laser energy of 40 µJ. The fragments point to **B)** PE 16:0/22:6 and PS 34:0 (blue). Position of the double bonds was not determined, neither the chains for the PS.

A.

B.

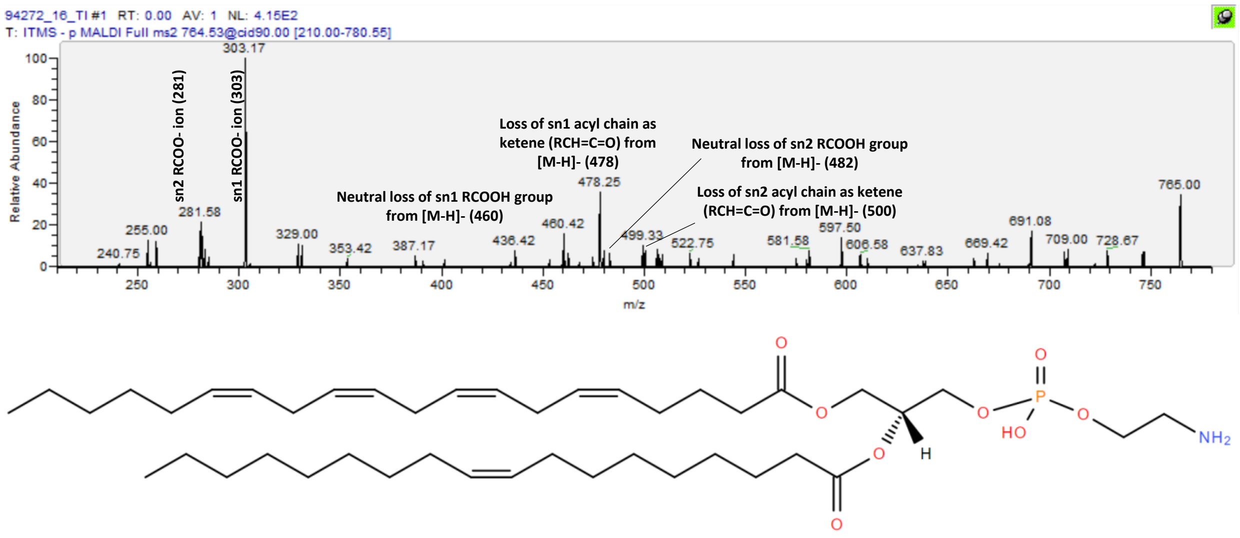

Figure S13. A) Fragmentation spectrum of m/z = 764.526 in negative polarity using a laser energy of 40 µJ. The fragments point to **B)** PE 20:4_18:1. Position of the double bonds was not determined.

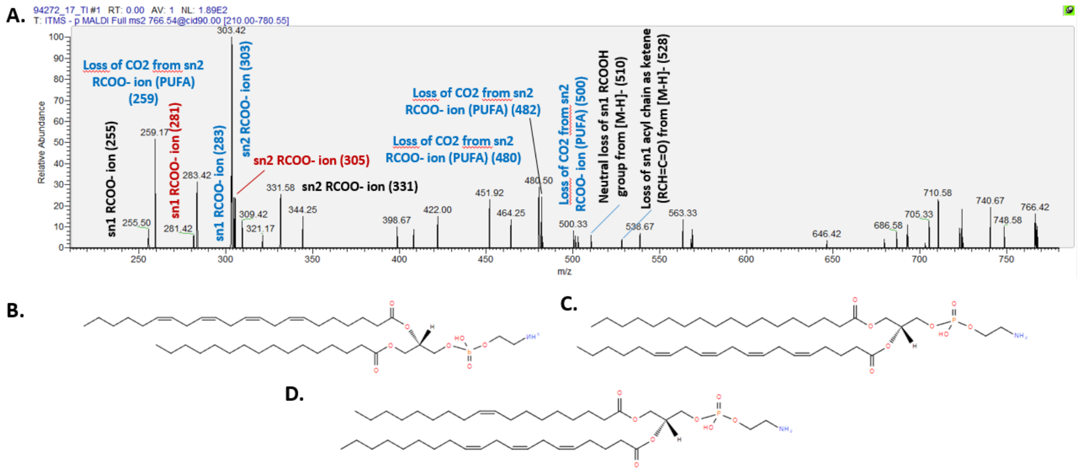

Figure S14. A) Fragmentation spectrum of m/ z= 766.537 in negative polarity using laser energy of 40 µJ. The fragments point to **B)** PE 16:0/22:4, **C)** PE 18:0/20:4 (blue) and **D)** PE 18:1/20:3 (red). Position of the double bonds was not determined.

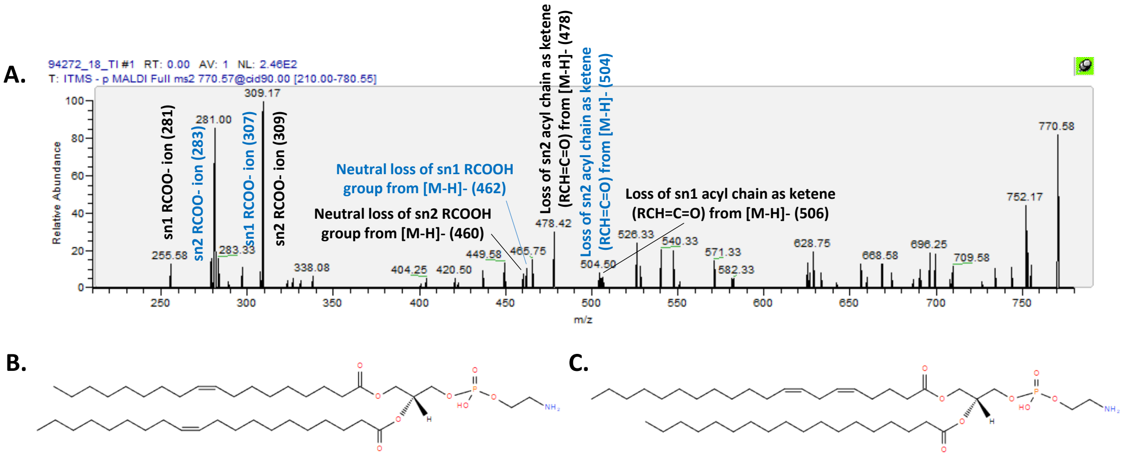

Figure S15. A) Fragmentation spectrum of m/z = 770.572 in negative polarity using laser energy of 40 µJ. The fragments point to **B)** PE 18:1_20:1 and **C)** PE 20:2_18:0 (blue). Position of the double bonds was not determined.

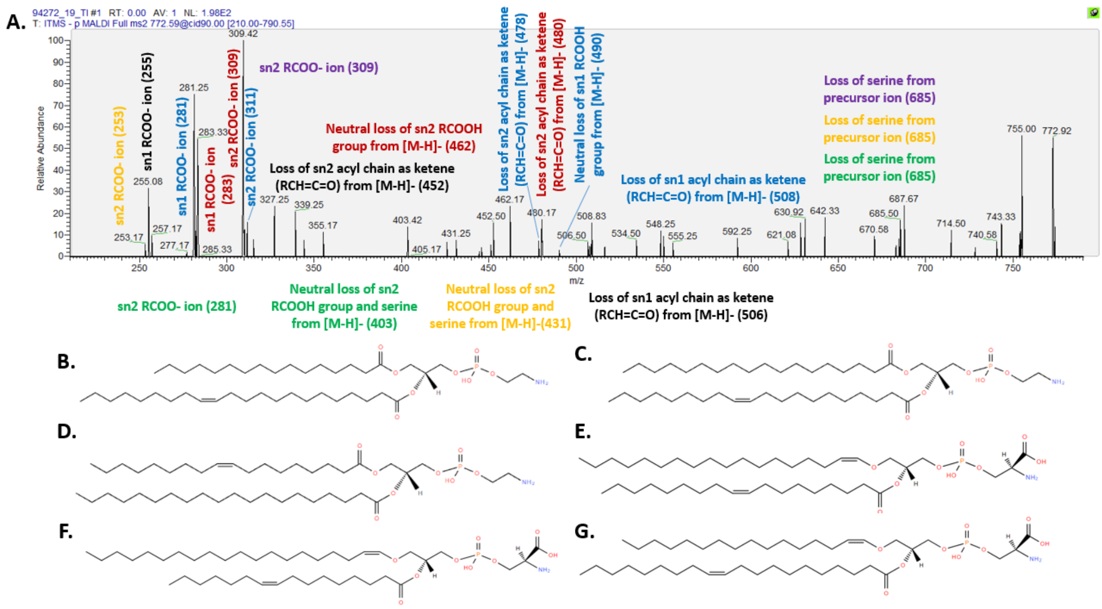

Figure S16. A) Fragmentation spectrum of m/z = 772.587 in negative polarity using laser energy of 40µJ. The fragments point to **B)** PE 16:0/22:1, **C)** PE 18:0/20:1 (blue), **D)** PE 18:1/20:0 (red), **E)** PS P-18:0/18:1 (green), **F)** PS P-16:1/20:0 (yellow) and **G)** PS P-20:1/16:0 (purple). Position of the double bonds was not determined.

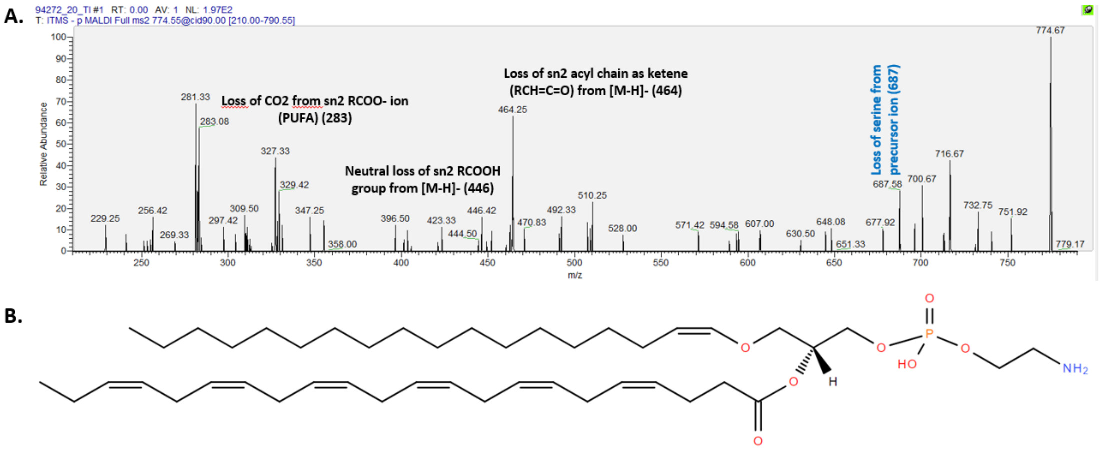

Figure S17. A) Fragmentation spectrum of m/z 774.547 in negative polarity using laser energy of 40 µJ. **A.** The fragments point to **B)** PE P-18:0/22:6 and PS 35:1 (blue). Position of the double bonds was not determined.

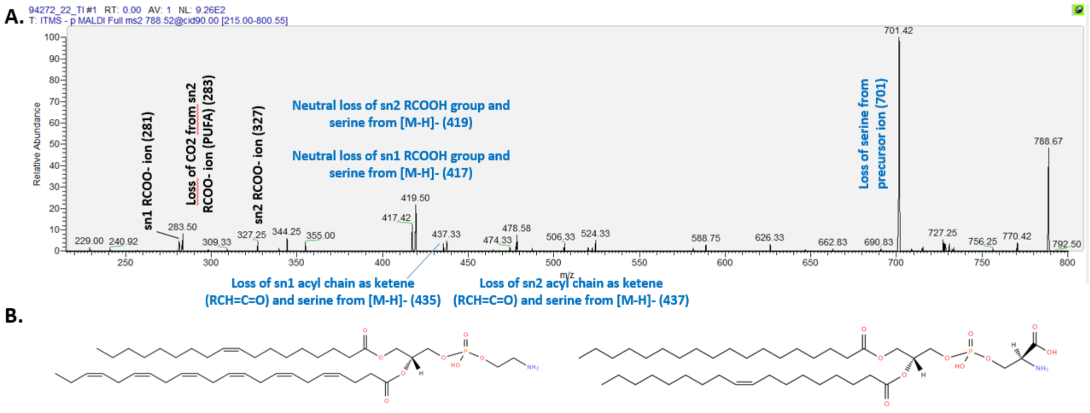

Figure S18. A) Fragmentation spectrum of m/z = 788.520 in negative polarity using laser energy of 40 µJ. The fragments point to **B)** PE 18:1/22:6 and **C)** PS 18:0_18:1 (blue). Position of the double bonds was not determined.

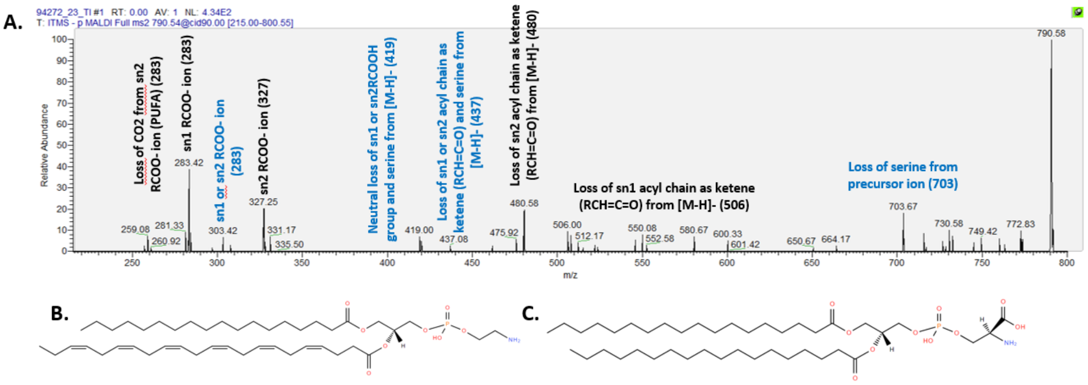

Figure S19. A) Fragmentation spectrum of m/z = 790.539 m/z in negative polarity using laser energy of 40 µJ. The fragments point to **B)** PE 18:0/22:6 and **C)** PS 18:0/18:0 (blue). Position of the double bonds was not determined.

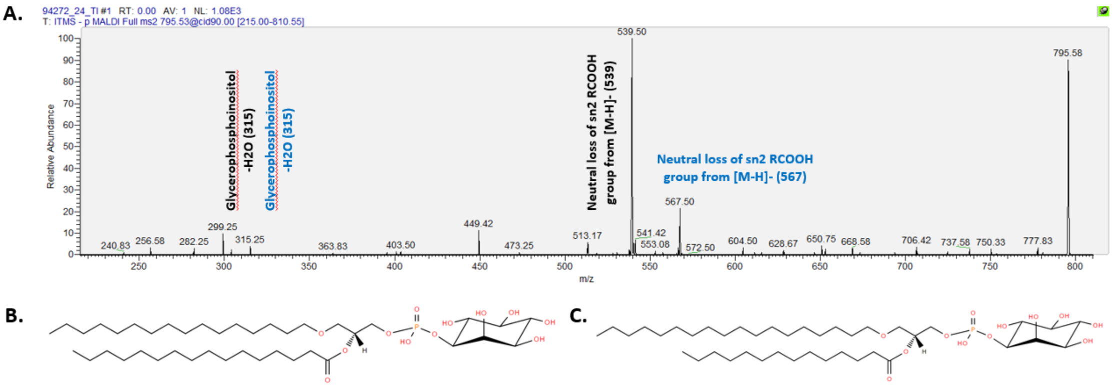

Figure S20. A) Fragmentation spectrum of m/z = 795.529 in negative polarity using laser energy of 40 µJ. The fragments point **B)** PI O-16:0/16:0 and **C)** PI O-18:0/14:0 (blue). Position of the double bonds was not determined.

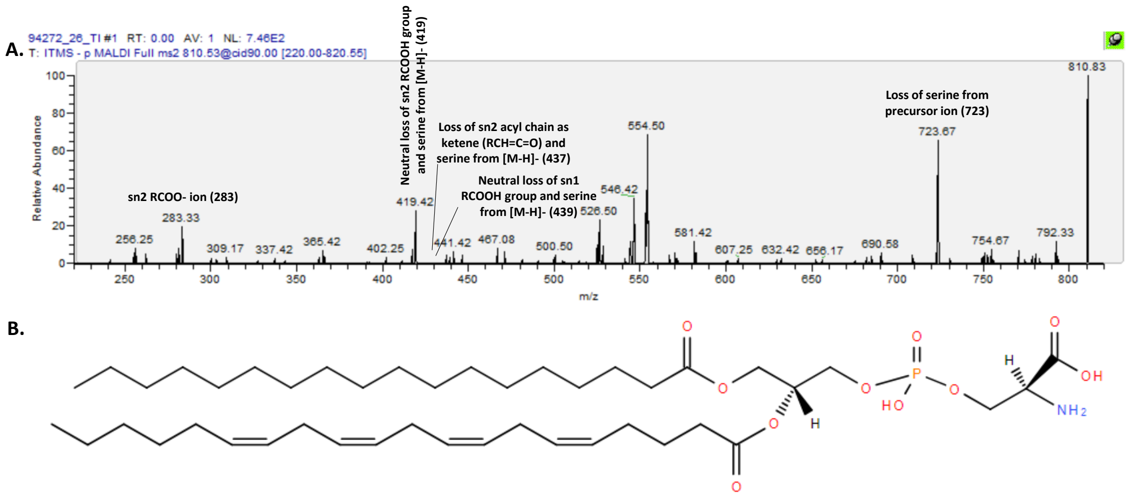

Figure S21. A) Fragmentation spectrum of m/z = 810.530 in negative polarity using laser energy of 40 µJ. The fragments point to **B)** PS 18:0/20:4. Position of the double bonds was not determined.

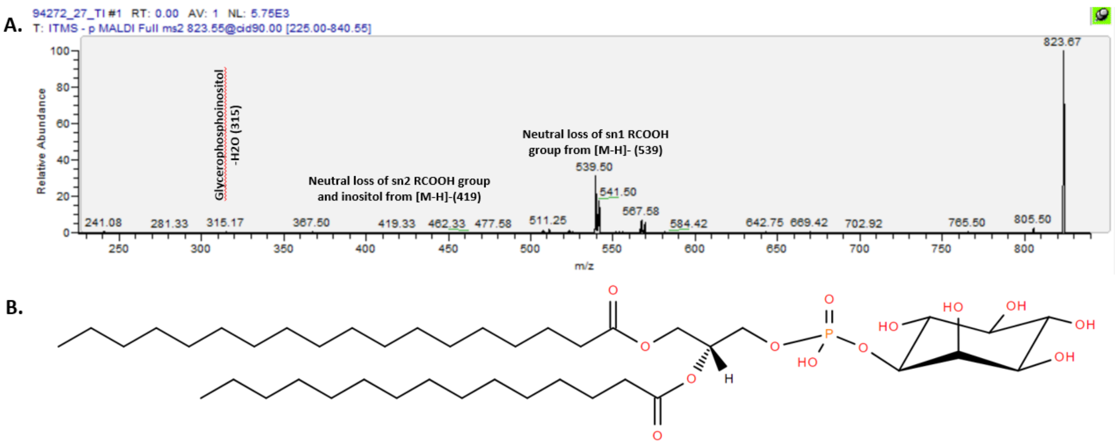

Figure S22. A) Fragmentation spectrum of m/z = 823.546 in negative polarity using laser energy of 40 µJ. The fragments point to **B)** PI 18:0_15:0. Position of the double bonds was not determined.

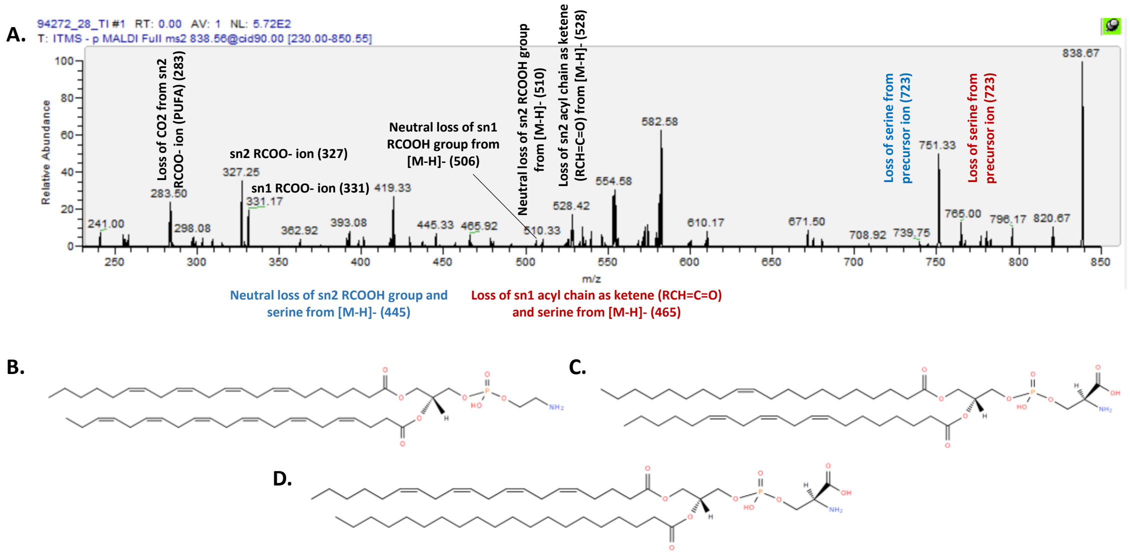

Figure S23. A) Fragmentation spectrum of m/z = 838.557 in negative polarity using laser energy of 40 µJ. The fragments point to **B)** PE 22:4/22:6, **C)** PS 20:1_20:3 (blue) and **D)** PS 20:0_20:4 (red). Position of the double bonds was not determined.

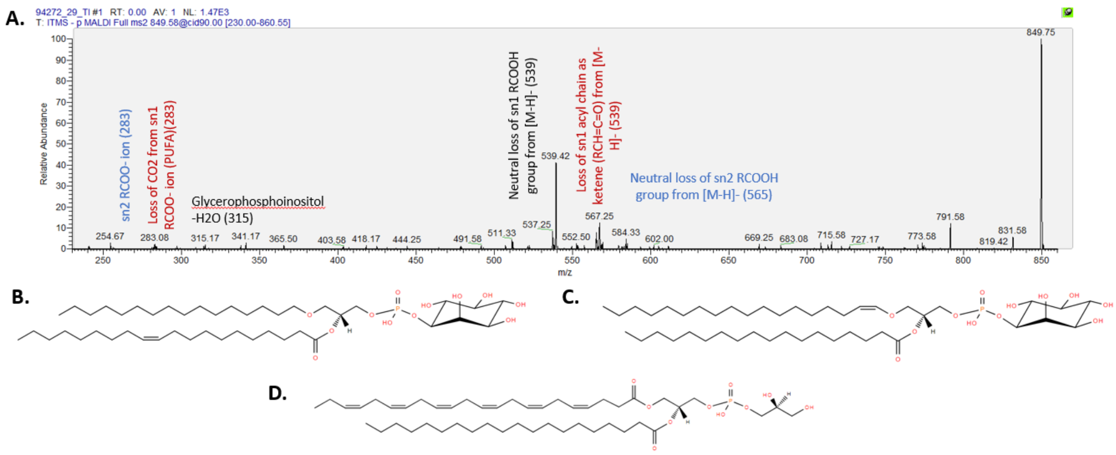

Figure S24. A) Fragmentation spectrum of m/z = 849.578 m/z in negative polarity using laser energy of 40 µJ. The fragments point to **B)** PI O-16:0_20:1, **C)** PI P-18:0/18:0 (blue) and **D)** PG 22:6_20:0 (red). Position of the double bonds was not determined.
